# Implications of the COVID-19 pandemic in eliminating trachoma as a public health problem

**DOI:** 10.1093/trstmh/traa170

**Published:** 2021-01-15

**Authors:** Seth Blumberg, Anna Borlase, Joaquin M Prada, Anthony W Solomon, Paul Emerson, Pamela J Hooper, Michael S Deiner, Benjamin Amoah, T Déirdre Hollingsworth, Travis C Porco, Thomas M Lietman

**Affiliations:** Francis I Proctor Foundation, University of California, San Francisco, San Francisco, CA, USA; University of Oxford, Oxford, UK; Faculty of Health and Medical Sciences, University of Surrey, Guildford, UK; Department of Control of Neglected Tropical Diseases, World Health Organization, Geneva, Switzerland; International Trachoma Initiative, Task Force for Global Health, Decatur, GA, USA; International Trachoma Initiative, Task Force for Global Health, Decatur, GA, USA; Francis I Proctor Foundation, University of California, San Francisco, San Francisco, CA, USA; Lancaster Medical School, Lancaster University, Bailrigg, Lancaster, UK; University of Oxford, Oxford, UK; Francis I Proctor Foundation, University of California, San Francisco, San Francisco, CA, USA; Department of Epidemiology and Biostatistics, University of California, San Francisco, San Francisco, CA, USA; Francis I Proctor Foundation, University of California, San Francisco, San Francisco, CA, USA; Department of Epidemiology and Biostatistics, University of California, San Francisco, San Francisco, CA, USA; Institute for Global Health Sciences, University of California, San Francisco, San Francisco, CA, USA; Department of Ophthalmology, University of California, San Francisco, San Francisco, CA, USA

**Keywords:** control, COVID-19, elimination, mass drug administration, mathematical modelling, trachoma

## Abstract

**Background:**

Progress towards elimination of trachoma as a public health problem has been substantial, but the coronavirus disease 2019 (COVID-19) pandemic has disrupted community-based control efforts.

**Methods:**

We use a susceptible-infected model to estimate the impact of delayed distribution of azithromycin treatment on the prevalence of active trachoma.

**Results:**

We identify three distinct scenarios for geographic districts depending on whether the basic reproduction number and the treatment-associated reproduction number are above or below a value of 1. We find that when the basic reproduction number is <1, no significant delays in disease control will be caused. However, when the basic reproduction number is >1, significant delays can occur. In most districts, 1 y of COVID-related delay can be mitigated by a single extra round of mass drug administration. However, supercritical districts require a new paradigm of infection control because the current strategies will not eliminate disease.

**Conclusions:**

If the pandemic can motivate judicious, community-specific implementation of control strategies, global elimination of trachoma as a public health problem could be accelerated.

## Introduction

Trachoma remains a major cause of preventable blindness, particularly in sub-Saharan Africa. Substantial reduction in the global prevalence of trachoma has been achieved, but the coronavirus disease 2019 (COVID-19) pandemic has caused unprecedented disruption of public health programs that combine surveillance of disease transmission with treatment for endemic districts. Since transmission cannot be measured directly, the World Health Organization (WHO) recommends monitoring trachoma by assessing the prevalence of trachomatous inflammation–follicular (TF) in the upper tarsal conjunctiva of children 1–9 y of age.^1,2^ For elimination of trachoma as a public health problem (termed trachoma ‘control’ herein), the WHO requirements include that the prevalence of TF in children be reduced to <5% in each formerly endemic district. One cornerstone of trachoma control is annual mass drug administration (MDA) of azithromycin to endemic districts.^3^ The pandemic is delaying regular MDA and thus allowing possible resurgence of active trachoma in some districts.

Trachoma is caused by repeated conjunctival infection with ocular strains of *Chlamydia trachomatis*.^4^ The bacterium is spread by direct contact from infectious individuals, fomites or flies that land on human eyes.^5^ Repeated infection causes inflammation of the conjunctiva that may then progress to scarring, subsequent in-turning of the eyelid and eventual irreversible destruction of the cornea.^6^ Fortunately there has been considerable progress towards elimination of trachoma as a public health problem, in line with targets set and partnerships fostered within the WHO Alliance for the Global Elimination of Trachoma by the year 2020 (GET2020). A strategy of surgery for advanced disease, mass administration of antibiotics, facial cleanliness and environmental improvement (the SAFE strategy) has been advocated by the GET2020 alliance. This approach is supported by randomized clinical trials that have demonstrated the efficacy of antibiotic therapy for reducing the prevalence of infection and inflammatory disease and the efficacy of surgery to reposition damaged eyelids.^7,8^ Because of these interventions, as well as the possible indirect benefit of other public health measures, 10 countries have now been validated by the WHO as having eliminated trachoma as a public health problem.^9^

Based on their intensity of transmission, we can categorize districts as subcritical, MDA-subcritical or supercritical. We delineate these levels of transmission by the basic reproduction number, R_0_, and the treatment-associated reproduction number, R_T_. We define R_0_ as the mean number of secondary infections each infection causes without intervention and in the absence of immunity of close contacts and R_effective_ as the mean number of secondary infections each infection causes in the presence of immunity.^10^ We define R_T_ as the mean number of new infections caused by each case in a district that is receiving annual, community-wide MDA with azithromycin. We define subcritical districts as those with R_0_<1 and thus control of trachoma would be anticipated regardless of MDA. We define MDA-subcritical districts as those with R_0_>1, but R_T_<1. These districts are progressing towards control, but only because of ongoing annual MDA. Finally, we define supercritical districts as those where both R_0_ and R_T_ are >1. These districts are not expected to achieve control, despite ongoing annual MDA. Our definitions of subcritical, MDA-subcritical and supercritical transmission roughly correspond to how other manuscripts describe hypoendemic, mesoendemic and hyperendemic settings, respectively.^11^ Here we utilize a mathematical model of trachoma transmission to evaluate how the disruption of MDA due to the COVID-19 pandemic may delay progress towards trachoma control.

## Methods

**Figure 1.**
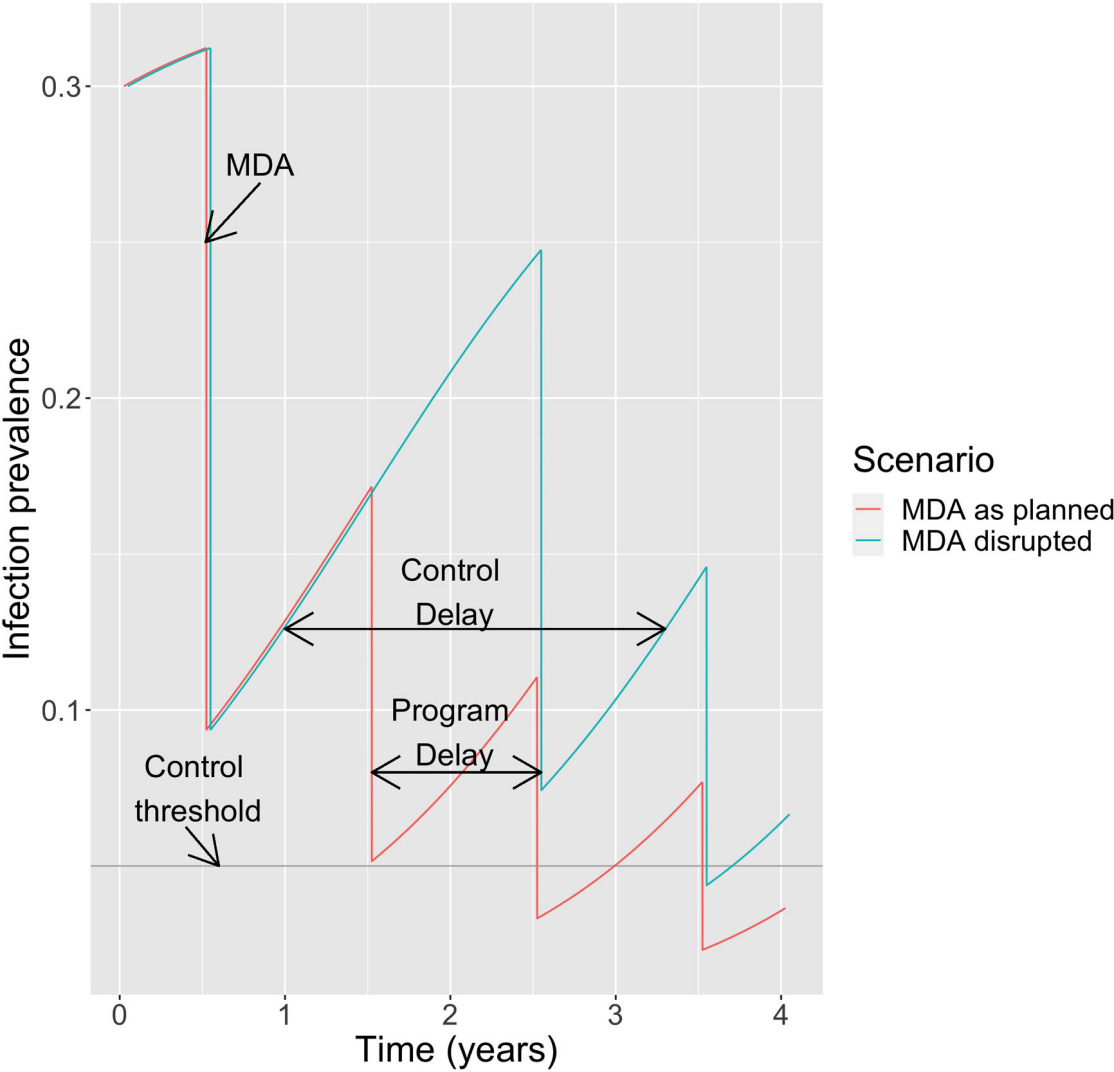
Schematic of the model. The ‘MDA as planned’ scenario involves periods of exponential growth of prevalence punctuated by uniformly spaced reduction due to annual MDA. The ‘MDA disrupted’ scenario assumes disruption of MDA programs occurs at year 1.0 and includes one missed round of MDA at year 1.5. Infection prevalence represents the proportion of children 1–9 y of age with current infection. Note that infection prevalence is distinct from the clinical manifestation of trachomatous inflammation–follicular. An R_0_ of 1.5 is assumed. The program delay is the length of MDA disruption. The control delay is the expected delay in trachoma control due to disruption of MDA. The horizontal grey line represents the 0.05 benchmark for trachoma control that aligns with the WHO's active trachoma criterion for elimination of trachoma as a public health problem.

**Figure 2.**
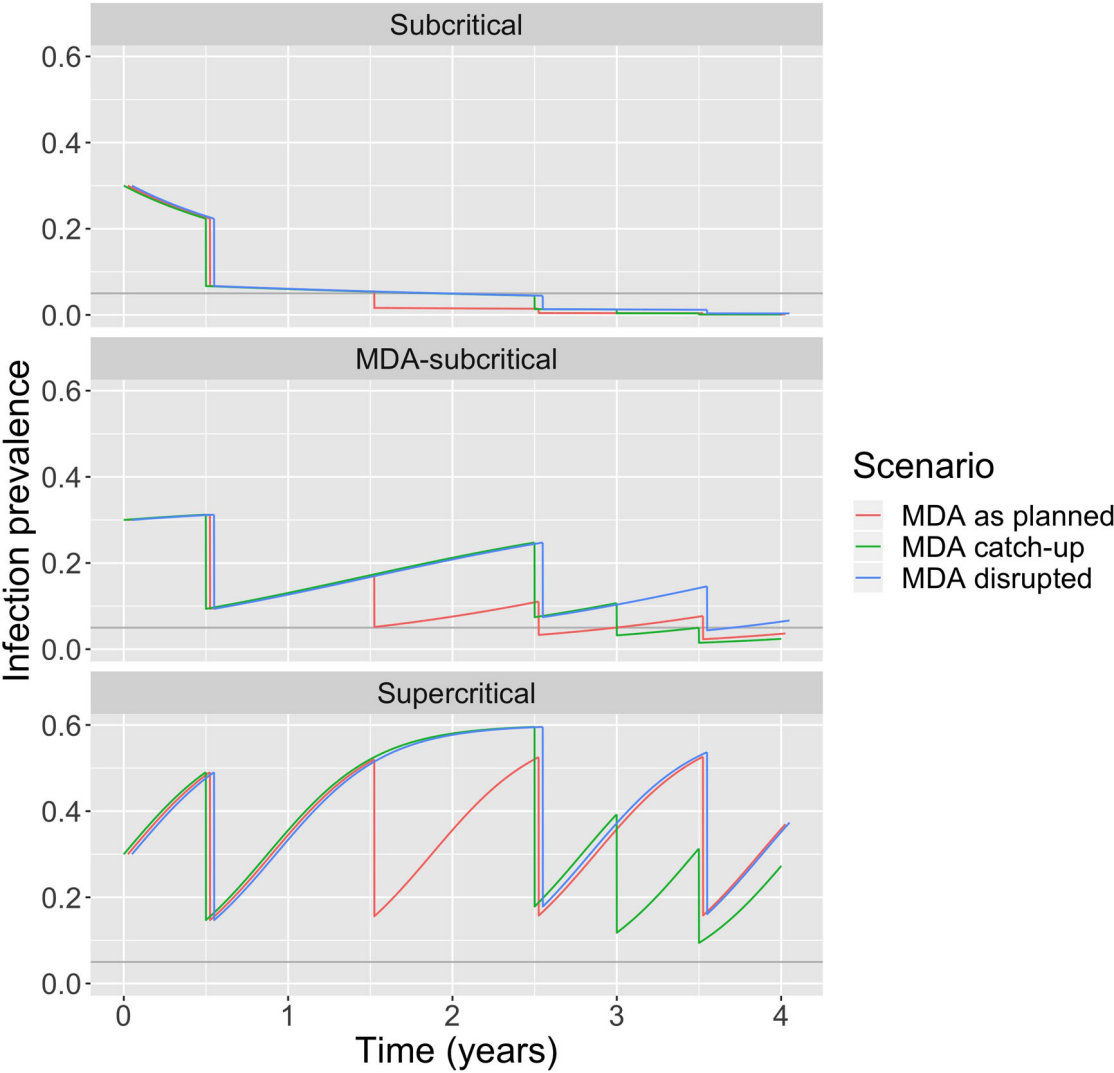
Modelling scenarios. Each panel corresponds to a different level of transmission, as defined by whether R_0_ and R_T_ are >1 or <1 (Table 1). The layout of each panel is similar to Figure 1. Within each panel the ‘MDA as planned’ scenario corresponds to no disruption of annual MDA. The ‘MDA disrupted’ scenario corresponds to a 1-y disruption starting at year 1.0 and includes skipping one annual MDA cycle at year 1.5. The ‘MDA catch-up’ scenario involves giving an extra MDA at year 3 after skipping an annual MDA at year 1.5. Between MDA cycles, the transmission dynamics are determined by a susceptible–infected–susceptible model (see Methods for details). For visual clarity, the time series corresponding to the scenarios are slightly offset horizontally. An R_0_ of 0.95, 1.5 and 2.5 are assumed for subcritical, MDA-subcritical and supercritical transmission, respectively. At the time of MDA disruption (year 1.0), the infection prevalence of the three transmission scenarios is 0.06, 0.13 and 0.36, respectively. This corresponds to an R_effective_ of 0.89, 1.30 and 1.61, respectively.

**Figure 3.**
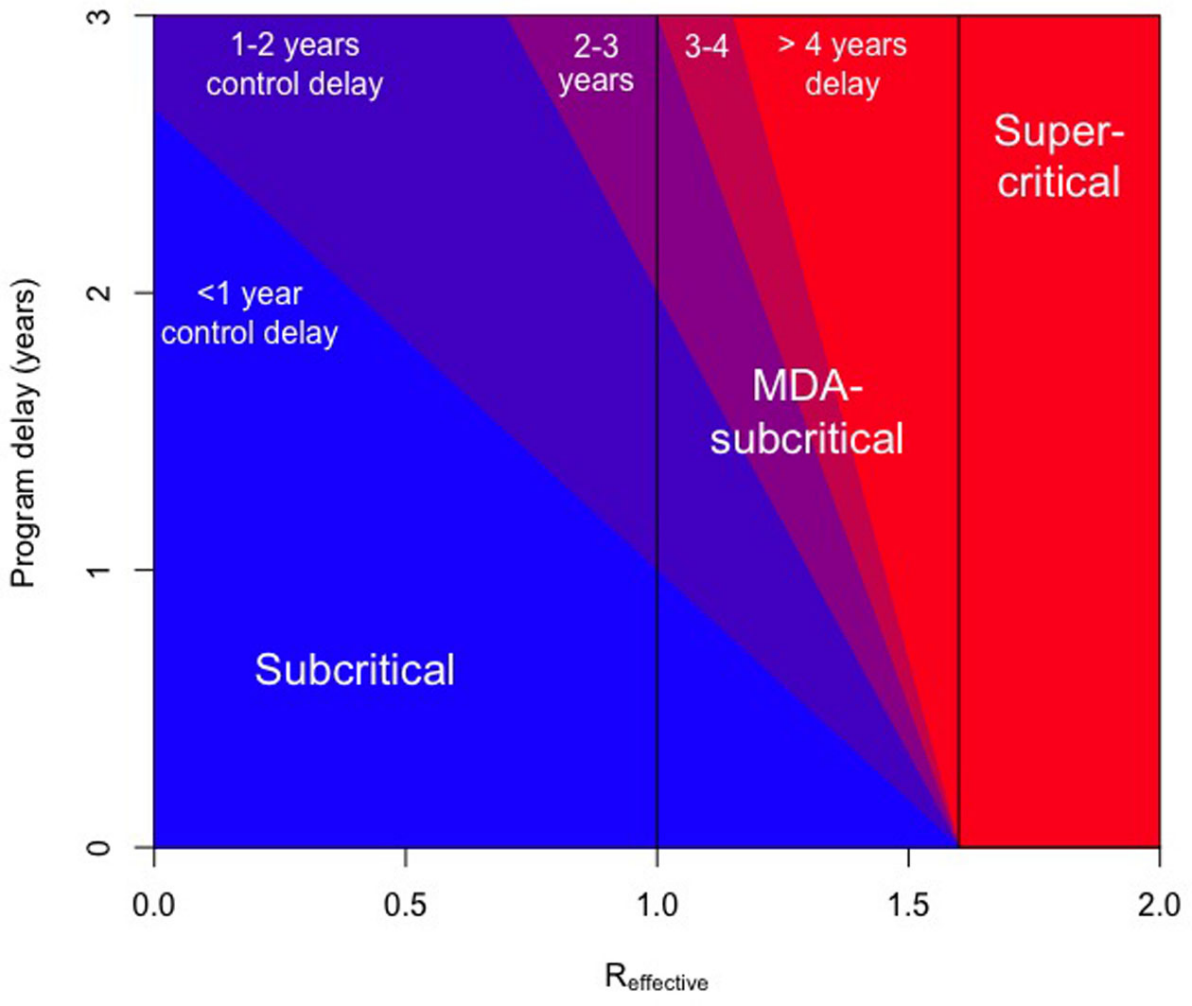
Our estimate of the control delay for trachoma is depicted by colour, based on the R_effective_ of the district and the program delay for the administration of MDA. The underlying model assumes annual MDA leads to a 70% decrease in trachoma incidence in the immediate post-treatment interval. The terms subcritical (R_effective_<1), MDA-subcritical (R_effective_>1–<1.6) and supercritical (R_effective_>1.6) refer to conditions in which infection is expected to be self-limited, requires annual MDA in order for control targets to be achieved or requires a new paradigm of treatment for eventual control, respectively. The different categories of transmission are demarcated by vertical black lines. Although our focus is on how the COVID-19 pandemic impacts trachoma control, the underlying model can be applied to a variety of diseases and program delay scenarios. For the subcritical and MDA-subcritical scenarios depicted in Figure 2 with a 1-y MDA disruption occurring at year 1.0, our estimate for the control delay is 0.85 and 2.02 y, respectively. The control delay is not calculable for the supercritical scenario because control is not expected to be achieved with annual MDA.

To simulate the prevalence of trachoma infection and estimate the delay in control caused by the COVID-19 pandemic we developed a simple model for trachoma transmission among children. We assume that children form a core group for transmission and that transmission from adults to children is negligible.^12^ Although the relative importance of different routes of transmission are still debated, it is commonly accepted that infection occurs by transfer of ocular *C. trachomatis* between uninfected children and infected children.^13,14^ Thus transmission can be modelled according to the mass-action assumption of the classical susceptible-infected model. In particular, infection prevalence is measured in children only and new infections occur at a rate that is proportional to the product of the fraction of children that are susceptible to infection and the fraction of children infected.^15,16^ Recovery from infection occurs at a rate that is proportional to the prevalence of infection. Once an infection clears, children become susceptible to infection again. Whenever a district receives a round of MDA, the number of infected people is reduced by the ‘overall MDA efficacy’, which we define as the product of azithromycin coverage and the probability that azithromycin clears infection from an individual. For Figures 1–3, we assume that the average duration of infection for children is 6  months, the antibiotic coverage is 80% and the azithromycin clearance is 87.5%.^16^

Based on the preceding susceptible–infected–susceptible model, trachoma control efforts are represented by periods of exponential growth (or decay) of infection that are separated by periodic reductions in infection due to annual MDA (Figure 1, red curve). The direction of exponential growth or decay is dependent on R_0_, with values of R_0_<1 indicating decay and R_0_>1 indicating growth. The specific exponential rate depends on R_effective_, which is the product of R_0_ and the proportion of the population that is not infected and not immune. We define the program delay as the time that an MDA cycle is delayed due to circumstances such as the COVID-19 pandemic (Figure 1, aqua curve). We define the control delay as the time gap between the beginning of MDA disruption and a return to the prevalence of infection prior to the disruption. By reflecting the additional time it will take for district-level control to be achieved, the control delay represents the overall consequence of a program delay.

We consider three scenarios for MDA. In the scenario labelled ‘MDA as planned’, one distribution of MDA occurs each year. In the scenario labelled ‘MDA disrupted’, one of the annual MDA cycles is missed. In the scenario labelled ‘MDA catch-up’, a cycle of annual MDA is missed and then a subsequent year has an additional MDA. We term this additional MDA as a ‘catch-up MDA’.

When using our model to estimate the control delay, we make the approximation that the change in prevalence between MDA time points follows an exponential curve. This is equivalent to assuming that the number of susceptible individuals is constant rather than that the entire population is constant. We acknowledge that this assumption does not hold well for districts that are close to equilibrium, such as supercritical districts or districts that have not yet received MDA. See the supplementary material (Text S1) for more methodological details.

## Results

The impact of a 1-y program delay differs depending on whether transmission is subcritical, MDA-subcritical or supercritical (Figure 2 and Table 1). In subcritical districts, the control delay is less than the program delay since prevalence is decreasing even in the absence of annual MDA. In MDA-subcritical districts, the control delay is longer than the program delay, but resumption of annual MDA cycles will put a district back on track for eventual control. An additional catch-up MDA would return a district to its original time course, resulting in a control delay close to zero. In supercritical districts, a program delay followed by resumption of the regular MDA schedule would be roughly equivalent, but not lead to control. In contrast, a program delay followed by a catch-up MDA could accelerate control in supercritical districts.

**Table 1.** Characteristics of subcritical, MDA-subcritical and supercritical transmission

Transmission	R_0_	R_T_	Control delay	Catch-up MDA restores progress
Subcritical	<1	<1	Less than program delay	Yes
MDA-subcritical	>1	<1	Greater than program delay	Yes
Supercritical	>1	>1	Not applicable, as control will not be achieved	Yes

Our model predicts that a program can get back on track if multiple MDA cycles are missed, provided that extra MDA cycles can be provided in subsequent years. For example, a program could get back on track after missing two annual MDA cycles if it had the resources to provide semi-annual MDAs for 2 y before resuming annual MDA. The exact timing of catch-up MDA does not matter. For example, the extra treatments could be 1, 3 or even 6 months after the scheduled annual MDA (supplementary materialText S1).

Our model predicts the control delay increases as the program delay or R_effective_ increases (Figure 3). Since R_effective_ decreases as the infection prevalence increases, this implies that the control delay is higher when MDA disruption occurs after a district has had multiple rounds of treatment. For our estimates of MDA coverage and efficacy, the threshold value for R_effective_ that differentiates MDA-subcritical and supercritical transmission is 1.6. As R_effective_ approaches that critical point, the duration of the control delay becomes increasingly large. The theoretical delay becomes infinite for supercritical transmission. This is a reflection that supercritical districts will require new treatment strategies in order to achieve trachoma control, even had there not been a disruption in the annual MDA schedule.

Although the prevalence of TF in any given year does not provide a direct measurement of R_effective_, R_0_ or R_T_, the trend of TF over time is related to these values.^17–20^ An important implication for TF surveillance is that our model of infection prevalence does not reflect the time course of TF itself. This is because TF is not a perfect marker of current infection, but rather a lagging indicator caused by the inflammatory response to infection.^21,22^ Thus TF prevalence surveys may not register the impact of decreased infection prevalence for 6–12 months.^19,20,23,24^

Estimates of the duration of infection in children have ranged from <10 weeks to >1 y.^15,16^ In addition the overall efficacy of MDA to decrease infection in the immediate post-treatment period is unknown. Sensitivity analyses show that for subcritical districts, the control delay increases as the duration of infection increases (supplementary material Figure S1, top panel). In contrast, for MDA-subcritical districts, the control delay decreases as the duration of infection increases. The same qualitative features can be seen for the impact of the overall MDA efficacy on the control delay (supplementary material Figure S1, bottom panel).

## Discussion

Our model provides a quantitative perspective on how trachoma control is impacted by MDA disruption as a consequence of the COVID-19 pandemic. Interpretation of our results in the context of findings from randomized controlled trials (RCTs) provides a framework for strategizing the effective distribution of future MDA. Recommended strategies for maintaining progress towards trachoma control vary based on whether a region is subcritical, MDA-subcritical or supercritical.

In subcritical districts, infection is difficult to detect, and RCTs have shown that trachoma may be controllable without multiple rounds of MDA.^25–27^ The empirical observation of self-contained transmission in subcritical districts is consistent with our model's prediction that a program delay in MDA will not lead to a noticeable delay for district-level control. The rare apparent resurgence reported from a few subcritical districts may be attributable to a combination of misclassification and measurement error.^14,28,29^

In MDA-subcritical districts, our model suggests that the delay in achieving control will be longer than the program delay. Our model is also consistent with other modelling studies that indicate a missed cycle of MDA can be mitigated by a single catch-up MDA.^11,30^ In addition, the exact timing of the catch-up MDA does not significantly affect the performance.^31^

In supercritical districts, the control delay can vastly exceed any delay due to COVID-19. This finding aligns with prior trials and models that show infection returns more rapidly after MDA in supercritical districts.^14,32,33^ Some of these districts may eventually head towards control due to subtle reductions in disease transmission caused by changes in socio-economic factors. However, in the limited number of districts where TF remains >30% after a decade of MDA, our model suggests that transmission is so efficient that annual MDA will not lead to control even if there is no disruption to azithromycin distribution. Thus alternative strategies need to be considered for control to be achieved in the near future.^34^

To address the need for adjunctive treatment strategies, much emphasis has been placed on more frequent administration of MDA. The success of this approach has been demonstrated with an RCT that compared quarterly MDA for children with annual MDA.^35^ This trial also supported the result of models that suggest children are the core group for transmission, so that adjunctive treatment for adults is unnecessary.^14^ Meanwhile, the predictions of other models of more frequent MDA have not shown good agreement with trial data. For instance, one model predicted that a second MDA given soon after the first would be especially effective at clearing infection.^36^ However, this finding has yet to be verified empirically. In fact, three rounds of MDA with high antibiotic coverage in 3 weeks did not prevent infection from returning in the Egyptian arm of the Azithromycin in Control of Trachoma study.^37^ Additional models predict that repeated biannual distribution will reduce infection more rapidly than annual distribution, but trials have not provided compelling evidence to support this prediction.^38–40^ Another adjunctive strategy for trachoma control involves improving water, sanitation and hygiene (WASH). Models predict that if WASH measures are able to reduce transmission by at least 10%, there could be benefit.^36^ However, no trial has yet proven that WASH offers any benefit over annual MDA alone. Of note, some of the studies that failed to show a significant impact of adjunctive treatment may have been underpowered, since they were conducted prior to the wide availability of sensitive nucleic acid amplification tests for identifying current infection.

Limitations of our estimate of control delay include approximations that may bias the estimate of the control delay. First, our analysis ignores how the number of susceptible individuals saturates as the prevalence of infection increases (Figure 2). Although this may be reasonable as we approach control, this approximation overestimates the between-MDA growth of the infected population in high-prevalence settings. That is, the true control delay may actually be shorter than our model's prediction (as seen by how ‘MDA catch-up’ has slightly lower infection prevalence in later years than ‘MDA as planned’ in the MDA-subcritical panel of Figure 2). In addition, the underlying assumptions of our transmission model ignore many important aspects of trachoma pathophysiology and epidemiology. These include the heterogeneity in transmission due to variable bacterial load, heterogeneity in susceptibility due to variable host immunity and heterogeneity in contact among members of the population. These factors might lead to a control delay longer or shorter than the model prediction. A final consideration is that our model assumes instantaneous delivery of MDA, but logistical programmatic barriers can cause delays in drug delivery within districts. Some of these limitations can be addressed by stochastic models that incorporate more than one state of infection.^11,30^

The impact of geographic heterogeneity on trachoma control deserves careful consideration, although this issue is not new to the pandemic era. The analyses in this article have been described at the health district level, as this is the geographic level in which infection prevalence surveys are typically acquired. However, there may be districts in which there is significant village-level heterogeneity of transmission within the district. Such heterogeneity would lead to different control delays for different villages within a district. Meanwhile several studies have shown that within a village, exponential growth of infection is typical.^33,41^ Thus subvillage heterogeneity is probably negligible. This is in accordance with the expected mixing patterns of children, who are primarily responsible for spreading disease.

Despite the limitations of the model, we hope our results can be useful for stakeholders involved in trachoma control (Table 2).^42^ From a policy perspective, our results provide reassurance that the resumption of trachoma control is feasible. Further work is needed to classify districts as MDA-subcritical versus supercritical, so that district-level distribution of MDA can be optimized. Catch-up treatments may be beneficial in MDA-subcritical districts, but the exact timing will likely not matter. In addition, maintenance of pre-pandemic MDA coverage and efficacy should be prioritized over early resumption of MDA with suboptimal coverage. Meanwhile, supercritical districts will require an alternate treatment strategy to annual MDA.

**Table 2.** Summary of policy-relevant items for reporting models in epidemiology of neglected tropical diseases^42^

Policy relevant principle	Application to article	Location of specific detail
Stakeholder engagement	Study inspired by NTD Modelling Consortium, which maintains direct communication with the WHO, BMGF, ITI and other stakeholders	Discussion
Complete model documentation	A complete description of our model is provided. Code is available in a GitHub repository	Methods/supplement (available from https://github.com/sblumberg/trachoma—COVID_impact)
Complete description of data used	Model is parameterized by previously published results focused on the transmission dynamics of trachoma	Methods
Communicating uncertainty	Assumptions of the model that lead to uncertainty in the results are described	Discussion
	Sensitivity analyses for the duration of the infectious period and overall MDA efficacy are presented	Supplemental figure
Testable model outcomes	An alternative model is presented in this special collection. Given the unprecedented nature of the COVID-19 pandemic, no data are currently available for model validation	See Borlase et al.^11^

BMGF: Bill and Melinda Gates Foundation; ITI: International Trachoma Initiative.

## Conclusion

When considering the impact of the COVID-19 pandemic on efforts to control trachoma, districts can be classified as subcritical, MDA-subcritical or supercritical. In subcritical districts, no significant delay in achieving control goals is anticipated. In MDA-subcritical districts, control after a 1-y program delay can be achieved by either extending the duration of annual MDA distribution or by providing an additional catch-up round of MDA. Meanwhile, supercritical districts will require adjunctive treatments in order to reach control milestones. Although models have endorsed a variety of adjunctive treatments in supercritical districts, quarterly MDA for children remains the only intervention shown to be statistically superior in field trials.^35^ The COVID-19 pandemic may offer the opportunity to reassess strategies to achieve trachoma control in these districts.

## Supplementary Material

traa170_Supplementary_Files

## Data Availability

All input for models are based on cited references.
