# Supplementary figures and images for: Implications of the COVID-19 pandemic in eliminating trachoma as a public health problem
Source: Trans R Soc Trop Med Hyg. 2021 Jan 15;115(3):222–8. doi: 10.1093/trstmh/traa170 (PMC7928550; doi:10.1093/trstmh/traa170)

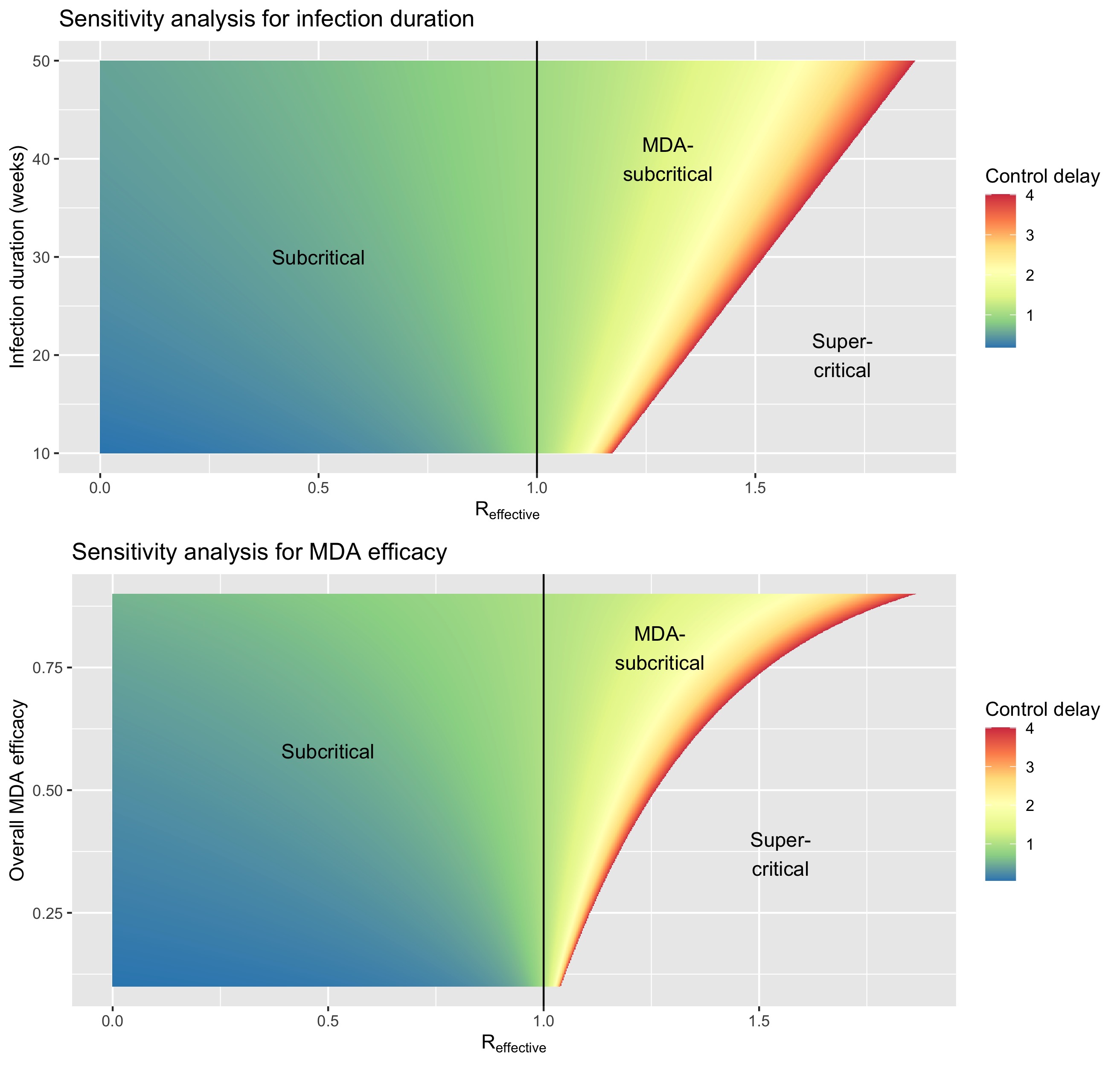

Supplement: traa170_Supplementary_Files [file traa170_supplementary_files.zip › Revised_Fig_S1-sensitivity.jpg]
